# Egg-inspired engineering in the design of thin-walled shelled vessels: a theoretical approach for shell strength

**DOI:** 10.3389/fbioe.2022.995817

**Published:** 2022-09-14

**Authors:** Valeriy G. Narushin, Michael N. Romanov, Darren K. Griffin

**Affiliations:** ^1^ Research Institute for Environment Treatment, Zaporozhye, Ukraine; ^2^ Vita-Market Ltd Zaporozhye, Zaporozhye, Ukraine; ^3^ School of Biosciences, University of Kent, Canterbury, United Kingdom

**Keywords:** avian egg, thin-walled shelled vessel, eggshell, neutral axis, egg shape model, Hügelschäffer’s model, Narushin’s model

## Abstract

A novel subdiscipline of bionics is emerging in the form of ‘egg-inspired engineering’ through the use of egg-shaped ovoids as thin-walled tanks and building structures. Hügelschäffer’s and Narushin’s models of egg geometry are highly applicable within this proposed subdiscipline. Here we conducted a comparative analysis between the two models with respect to some of the most important egg parameters. These included contents volume, shell volume, and the location of the neutral axis along the shell thickness. As a first step, theoretical studies using the Narushin’s model were carried out due to the lack (or limited amount) of data on the geometric relationships of parameters and available calculation formulae. Considering experimental data accumulated in the engineering and construction industries, we postulate a hypothesis that there is a correlation between location of the neutral axis and the strength of the walls in the egg-shaped structure. We suggest that the use of Narushin’s model is preferable to Hügelschäffer’s model for designing thin-walled shelled vessels and egg-shaped building structures. This is due to its relative simplicity (because of the requirement for only two initial parameters in the basic equation), optimal geometry in terms of material costs per unit of internal capacity, and effective prerequisites for shell strength characteristics.

## Introduction

### Egg-inspired engineering

Natural biological objects have repeatedly inspired scientists to develop unique engineering technologies. Direct copying of biological systems and their embodiment in metal, synthetic, building or other materials achieve surprising efficiency. Indeed, the field of bionics (the study of mechanical systems that function like (parts of) living organisms) has arisen as a result of this. Such copying can both lead and inspire human imagination in creating man-made structures. Specifically, bird egg shells have inspired numerous man-made mechanical structures.

Possessing an asymmetric profile, a relatively thin shell and a rather fragile structure, the eggshell nonetheless withstands high loads, holds a fairly voluminous content and, at the same time, is very effective in terms of the specific material costs required to achieve its design features. It is these geometrical and mechanical properties that have facilitated its increasing use in engineering solutions, specifically applied to designing thin-walled shells. Results of numerous studies of these structures (Lazarus et al., 2012; Zhang et al., 2017a,b, 2019, 2021, 2022; Guo et al., 2020 have highlighted these beneficial characteristics, i.e., high loading capacity, efficient space utilization, amazing weight-to-strength ratio, proper span-to-thickness ratio and streamlined forms.

In addition to use for constructing tanks and other thin-walled vessels, the popularity of eggshell shapes can also be seen in building structures (Freiberger, 2007; Petrović et al., 2011) and bridge design (Chen and Sha, 2005). Thus, here we propose a subdiscipline of bionics, which henceforth we refer to as ‘egg-inspired engineering.’

### Egg-shaped models

As a starting point, one can embody structurally a certain engineering object in the shape of an egg through use of its respective mathematical model. Despite fairly extensive research in this field for ∼70 years (e.g., Preston, 1953; Carter, 1968; Todd and Smart, 1984; Smart, 1991; Baker, 2002; Troscianko, 2014; Biggins et al., 2018; Pike, 2019), the Narushin’s (Narushin, 2001b) and Hügelschäffer’s (Petrović and Obradović, 2010; Petrović et al., 2011; Obradović et al., 2013) models have recently gained the most attention. While the former is thought to be more applicable in thin-walled shell structures (Zhang et al., 2017a; b, 2019, 2021, 2022), the latter has been more adapted to architectural and construction design (Petrović et al., 2011; Maulana et al., 2015).

*Narushin’s model* is an egg shape formula proposed by Narushin (2001b):

$$y=\pm \sqrt{L^{\frac{2}{n+1}}x^{\frac{2n}{n+1}}-x^{2}}$$ (1)

where *x* is the coordinate along the longitudinal axis and *y* the transverse distance to the egg profile, *L* is the egg length, and *n* is approximately defined from the following equation (Narushin, 1997):

$$\frac{B}{L}=2\sqrt{\frac{n^{n}}{{\left(n+1\right)}^{n+1}}}$$ (2)

where *B* is the maximum breadth of the egg.

Narushin (2001b) approximated Eq. 2 with a power law, substituting into it the values of *n* in the range from 1 to 6, which corresponded to the entire possible interval of the bird egg shape index, *B*/*L*, i.e., when *B*/*L* = [0.48 … 1]:

$$n=1.057{\left(\frac{L}{B}\right)}^{2.372}$$ (3)

*Hügelschäffer’s model* is named after the German engineer Fritz Hügelschäffer, who originally developed an oviform curve, shaped like an egg, by moving one of concentric circles along its *x*-axis to create an asymmetric ellipse (Ursinus, 1944; Schmidbauer, 1948; Ferréol, 2017). Petrović and Obradović (2010) derived a theoretical mathematical dependence for this curve, which we then modified in relation to the egg’s primary measures (i.e., its length, *L*, and maximum breadth, *B*) and carefully examined as applicable to chicken eggs (Narushin et al., 2020) as follows:

$$y=\pm \frac{B}{2}\sqrt{\frac{L^{2}-4x^{2}}{L^{2}+8wx+4w^{2}}},$$ (4)

where *w* is a further geometrical parameter that reflects the distance between two vertical axes that correspond to the egg’s maximal breadth and half of its length.

While Hügelschäffer’s model has been thoroughly investigated in terms of its geometric (Petrović and Obradović, 2010; Obradović et al., 2013; Narushin et al., 2020, 2021c, 2022b,c) and mathematical properties (Maulana et al., 2015; Narushin et al., 2020, 2021a,b, 2022a,b), this gap should be filled for Narushin’s model.

In no way detracting from the right to utilize both models, we, nevertheless, believe that it worthwhile to explore which of them is perhaps a more effective model for using in engineering and building structures. Obviously, there should be a certain factor (or several factors) that allow to turn the scales towards one or another mathematical function, i.e., Eqs. 1–4. Obviously, considering the performance indicators of egg-shaped structures, the one that will provide (i) the greatest strength, (ii) the best capacity and (iii) the lowest material costs for its manufacture will be optimal. Thus, the appropriate theoretical investigation should be carried out in such a way as to achieve provisions (i) to (iii) for each model. While indicators (ii) and (iii) can be characterized, by analogy with the parameters of a bird’s egg, as the shell volume (in other words, the material consumption of the vessel shell), *V*_*s*_, and volume of the interior (i.e., capacity of the vessel), *V*_*i*_, it is not so clear with a measure of strength (i). Based on our previous studies (Narushin et al., 2022c) on the geometric features of bird eggshells, however, the parameter (i) can be linked to the position of the neutral axis of the eggshell.

### Neutral axis

When the fibers are neither stretched nor compressed or when the longitudinal tension is zero, the neutral axis of eggshells and any bent geometric object is an imaginary line. The so-called ‘*k*-factor,’ which is a ratio of the position of the neutral axis to the thickness of the material, describes its precise placement. In other words, the equation for *k* is *k = t*/*T*, where *t* is the position of the neutral axis and *T* is the thickness of the material (Diegel, 2002). Importantly, *k* is not always equal to 0.5, meaning that the neutral axis does not always run through the exact center of *T*. Rather, *k* is influenced by the bend radius, material thickness, material characteristics and composition, and finally the forces used to bend the structure (Bernardo and Lopes, 2004; Betts et al., 2010; Kroes et al., 2013).

In construction and engineering, deep research has been carried out on the neutral axis location, i.e., concerning the *k*-factor value for various materials and structures. The relationships between *k* and strength characteristics have also been evaluated. To the best of our knowledge, the neutral axis location in the shells of closed reservoirs has not been analyzed, however it has been established that this parameter correlates with the load magnitude, as a result of which this indicator can be a predictor of loads (Mohammadhassani et al., 2011; Saqan and Rasheed, 2011; Sigurdardottir and Glisic, 2013; Lou et al., 2020; Aloupis et al., 2021). Nonetheless it is difficult to state unambiguously the role of neutral axis and how it will be observed in an unloaded closed structure with a large margin of strength. However, based on the observations that with increasing load, the neutral axis shifts into the depth of bending material (Bernardo and Lopes, 2004; Shim et al., 2009; Anastasopoulos et al., 2019; Bernardo et al., 2019; Vrijdaghs et al., 2021), it can be assumed that the higher the neutral axis, the greater the margin of strength in the analyzed structure. After all, the magnitude of its possible displacement is limited by the shell thickness. Therefore, the higher its initial location is relative to the surface, the greater the margin of its possible displacement to the inner edge can be allowed. Thus, it is probable that a comparative assessment of the depth of the neutral axis for various egg-shaped structures will reveal the strength potential of a particular mathematical egg model. That is, the closer the neutral axis runs to the outer surface, the more likely the egg-shaped vessel will be stronger (with all other parameters being equal).

Collectively, the objective of this study was to compare theoretically the two egg models, Narushin’s (Eq. 1) and Hügelschäffer’s (Eq. 4), to infer: (1) volume of the shell material of thin-walled vessels (as comparable to the eggshell volume), (2) volume of the vessels contents, and (3) the depth of the neutral line along the vessels shell.

## Materials and methods

Since the current study was purely theoretical in nature, two mathematical models served as material for comparative analysis, and their utilization made it possible to represent thin-walled closed shells shaped like a bird’s egg virtually. These were Narushin’s model (Eq. 1) and Hügelschäffer’s model (Eq. 4).

This comparative analysis involved the derivation of theoretical formulae enabling to calculate the volume of the shell material, or, following the professional terminology of the poultry industry, the eggshell volume, *V*_*s*_; the volume of egg contents, *V*_*i*_; and the parameter *k*. The latter describes the depth of the neutral axis inside the shell and equals the ratio *t*/*T*, where *t* is the distance from the shell surface to the place of the neutral axis’ conditional passage, and *T* is the shell thickness.

The methodical approach to infer the theoretical dependencies encompassed few fundamental formulae characterizing the egg geometry and included the following steps:

1. Define the egg volume, *V*, as a sum of the volumes of its constituents, i.e., egg contents volume, *V*_*i*_, and shell volume, *V*_*s*_:

$$V=V_{s}+V_{i}$$ (5)

**FIGURE 1:**
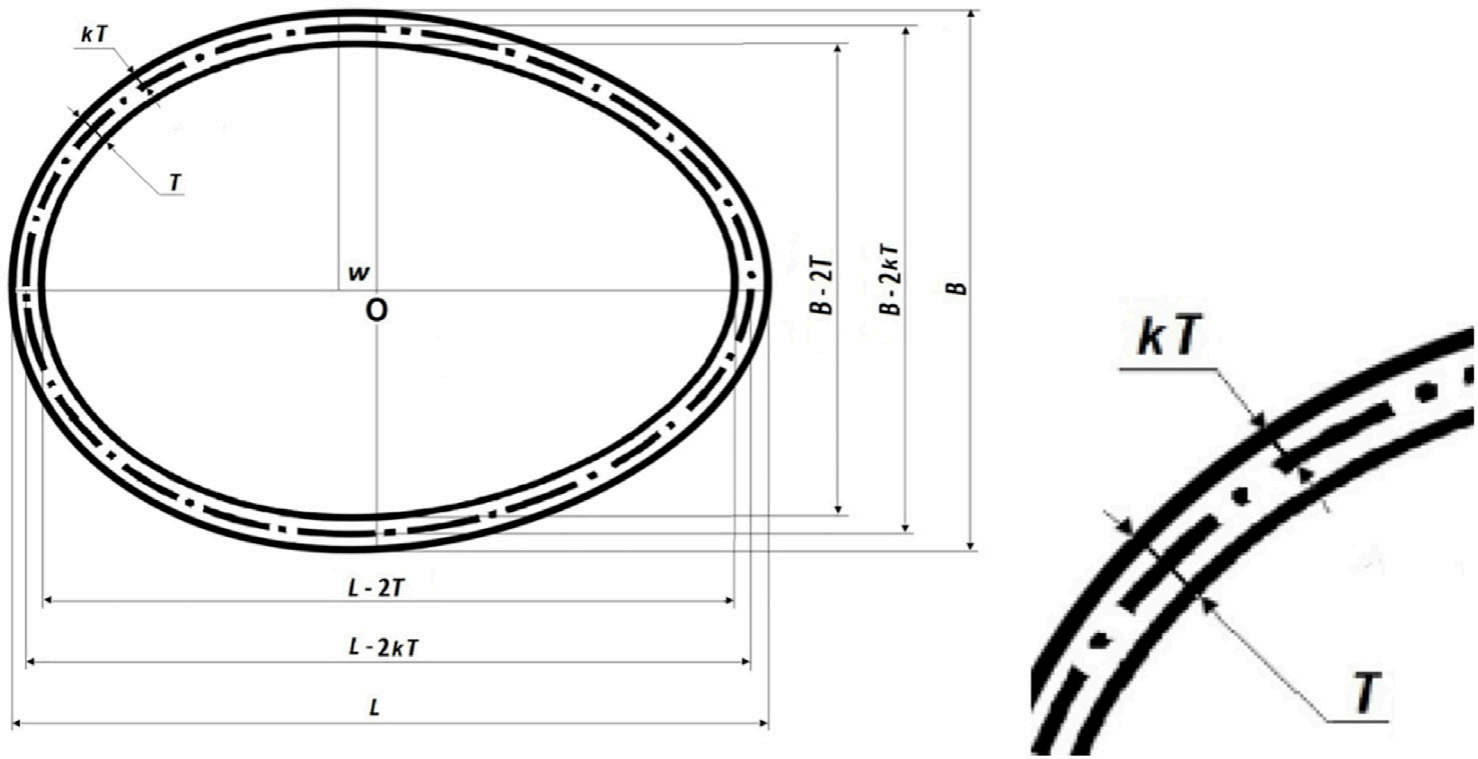
Geometrical interpretation of an egg-shaped ovoid with expanded portion (right) indicating the neutral axis *kT* (adapted from Narushin et al., 2021b).

2. Using the known formula for calculating *V*, determine *V*_*i*_ values by reducing the geometric dimensions (*L* and *B*) by twice the shell thickness, *T* (Figure 1, as also shown in Narushin et al., 2022c).
3. Calculate *V*_*s*_ as the difference between *V* and *V*_*i*_.
4. Use another approach to find *V*_*s*_, i.e., as the product of its surface area along the neutral axis, *S*_*n*_, and the thickness, *T*:

$$V_{s}=S_{n}\cdot T$$ (6)

5. Based on the known formula for calculating the surface area of an egg, *S*, derive a dependence for *S*_*n*_ by reducing the geometric dimensions (*L* and *B*) by twice the value of *kT* (Figure 1; Narushin et al., 2022c). As a result, a calculation formula for *k* can be obtained.

In the previous study (Narushin et al., 2022c), we carried out a similar theoretical procedure for the Hügelschäffer’s model that resulted in the following calculation formulae:

• Shell volume:

$$V_{s}=1.054T\left(B^{2}+2\left(B-T\right)\left(L-2T\right)-0.41\left(B-T\right)w\right)$$ (7)

• Contents volume:

$$V_{i}=0.527{\left(B-2T\right)}^{2}\left(L-2T-0.205w\right)$$ (8)

• *k* value that can be calculated from the following cubic equation:

$$k^{3}-\left(0.647\frac{B}{L}+0.853\right)\frac{L}{T}\cdot k^{2}+\left(\left(0.176+0.333\frac{B}{L}-0.011{\left(\frac{B}{L}\right)}^{2}\right){\left(\frac{L}{T}\right)}^{2}+\left(0.337\frac{B}{L}+0.167\right)\frac{L}{T}-0.337\right)k+$$

$$0.005{\left(\frac{B}{L}\right)}^{2}\cdot {\left(\frac{L}{T}\right)}^{3}-0.004\frac{B}{L}\cdot {\left(\frac{L}{T}\right)}^{3}-0.169\frac{B}{L}\cdot {\left(\frac{L}{T}\right)}^{2}-0.083{\left(\frac{L}{T}\right)}^{2}+0.169\frac{L}{T}=0$$ (9)

Therefore, the problem preceding the comparative analysis of two ovoid models is reduced to a deeper examination of the geometric parameters of the ovoid formed by rotating the contours of Narushin’s model (Eq. 1) around its horizontal axis.

**FIGURE 2:**
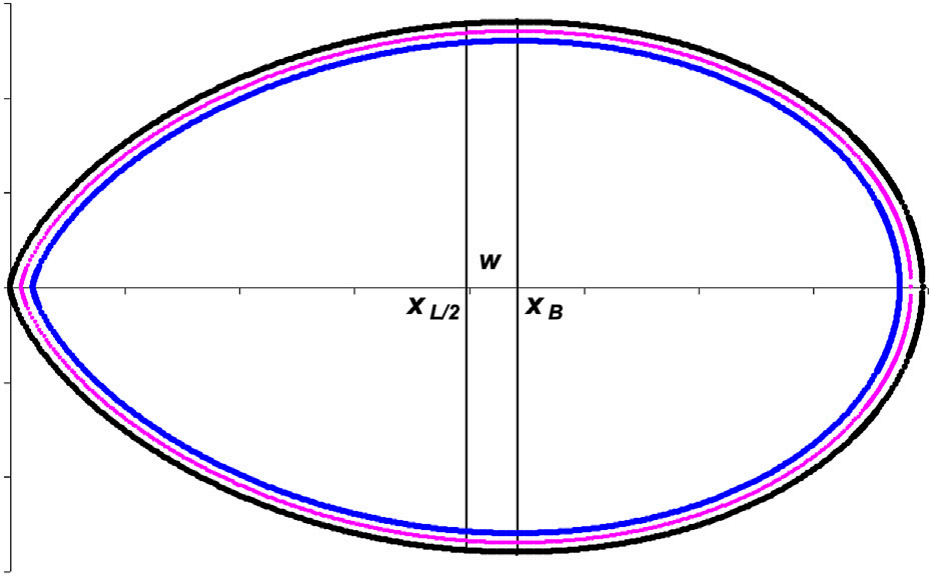
Geometrical interpretation of a vessel with a shape according to Narushin’s model.

To determine *V* and *S* of the resulting ovoid figure, the classical equations of integral geometry were used. Note that Eqs. 7–9 include the third parameter *w* as a key characteristic of Hügelschäffer’s model (Eq. 4). In contrast, Narushin’s model (Eq. 1) is described by only two linear dimensions, *L* and *B*. To bring both models to the same form, it is necessary to calculate what the value of the vertical axis shift (otherwise, the parameter *w*) for Eq. 1 corresponds to. To do this, we found the difference between the values on the *x*-axis conforming to (i) the maximum breadth of the ovoid, *x*_*B*_, and (ii) half of its length, *x*_*L*/2_ (Figure 2).

The results of implementing this methodology are outlined below in the next section.

## Results

### Mathematical interpretation of Narushin’s model

Since Narushin’s model has not been studied in sufficient depth in mathematical terms, we initially focused on filling this gap.

To establish how the dependence *n* = *f* (*B*/*L*) will change in a wider range, covering also values that exceed the range of actual bird eggs, we performed some transformations of Eq. 2:

$$4{\left(\frac{L}{B}\right)}^{2}=\left(n+1\right){\left(1+\frac{1}{n}\right)}^{n}$$ (10)

Based on Eq. 10, *n* values should be more than zero, i.e., *n* > 0. Then, n tending to 0 means a significant excess of *B* over *L*. On the contrary, when *n* tends to ∞, *L* is much greater than *B*.

**FIGURE 3:**
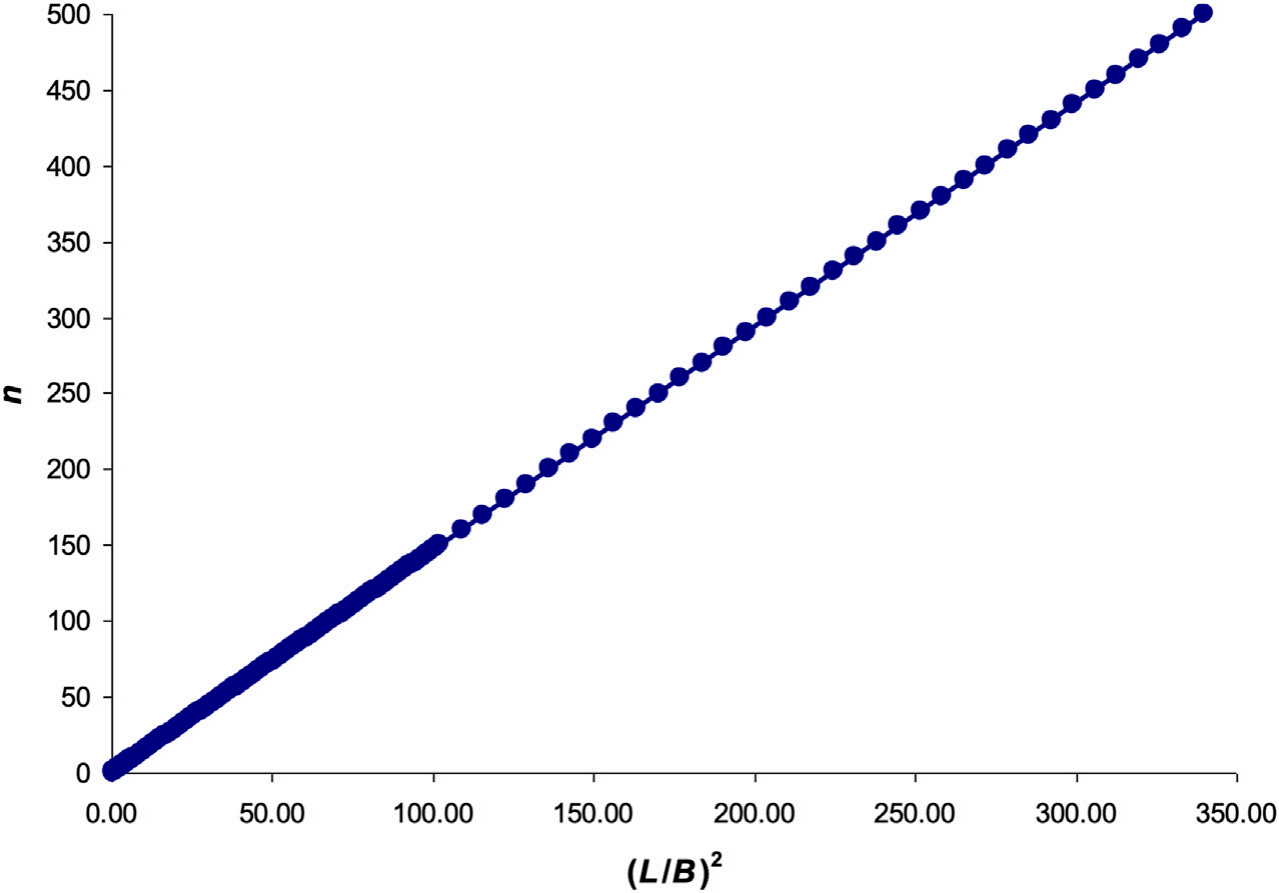
Graphical interpretation of Eq. 10.

Substituting *n* in Eq. 10 with the values from 0.01 to 500 (an arbitrary limit we chose), the graphical dependence shown in Figure 3 was obtained.

The resulting linear dependence was approximated by the following formula:

$$n=1.4715{\left(\frac{L}{B}\right)}^{2}-0.4915$$ (11)

*R* ^2^ = 0.99999999.

The produced Eq. 11 and the original Eq. 10 enabled us to theoretically generalize, to some extent, the results obtained due to the approximation. In particular, the limit to which the part of Eq. 10 tends is none other than Euler’s number, *e* ≈ 2.718282:

$$\underset{n\to \infty }{\lim}{\left(1+\frac{1}{n}\right)}^{n}=e$$ (12)

We tried to link Euler’s number, the number four in Eq. 10, and the constant coefficient 1.4715 in Eq. 11, resulting in a more harmonious relationship for *n* = *f* (*B*/*L*):

$$n=\frac{4}{e}\left({\left(\frac{L}{B}\right)}^{2}-\frac{1}{3}\right)$$ (13)

for which *R* ^2^ was also 0.99999999.

Thus, Eqs 11, 13 were taken as the basis for all our further calculations instead of the previous Eq. 3.

In the next step, we determined what the parameter *w* in Narushin’s model is equal to. According to the methodological assumptions and graphical interpretation (Figure 2),

$$w=x_{B}-x_{L/2}$$ (14)

where *x*_*B*_ is the value on the *x*-axis, corresponding to the maximum breadth of the ovoid, and *x*_*L*/2_ is the value on the *x*-axis, corresponding to half of its length.

The *x*_*B*_ value was derived by Narushin (2001b) and conforms to

$$x_{B}=L{\left(\frac{n}{n+1}\right)}^{\frac{n+1}{2}}$$ (15)

Then, the desired difference in Eq. 14 was transformed into the final dependence:

$$w=\frac{B\sqrt{n}-L}{2}$$ (16)

Substituting Eq 13 into Eq. 16, we obtained:

$$w=\frac{\sqrt{3L^{2}-B^{2}}}{\sqrt{3e}}-\frac{L}{2}$$ (17)

and, after substituting the value *e* ≈ 2.718282,

$$w=0.35\sqrt{3L^{2}-B^{2}}-0.5L$$ (18)

### Calculation of the external parameters of an ovoid described using Narushin’s model

In the previous investigation (Narushin, 2001b), the formulae for calculating the main external parameters of eggs (*V* and *S*) were inferred based on the proposed Narushin’s model. However, some formulae in that model involved approximate calculation. Also, considering a number of the above refined equations, we thought it expedient to recalculate the expressions for *V* and *S*.

According to Narushin (2001b), the basic formula for calculating the egg volume is as follows:

$$V=\frac{2\pi L^{3}}{3\left(3n+1\right)}$$ (19)

Considering the obtained Eq. 13, we transformed it into the following formula:

$$V=\frac{\mathrm{2\pi}e}{3}\cdot \frac{LB^{2}}{12+\left(e-4\right){\left(\frac{B}{L}\right)}^{2}}$$ (20)

and, substituting the numerical values of the constant coefficients,

$$V=\frac{4.44}{9.37-{\left(\frac{B}{L}\right)}^{2}}LB^{2}$$ (21)

Previously, Narushin (2005) demonstrated a linear relationship for the coefficient at *LB* ^2^ when calculating *V* using Eq. 1 of the Narushin’s mathematical model. Nevertheless, judging from Eq. 21, this coefficient has a clearly curvilinear character.

For comparative analysis, we rewrote Eq. 21 as follows:

$$V=K_{V}LB^{2}$$ (22)

where the coefficient *K*_*V*_ is equal to

$$K_{V}=\frac{4.44}{9.37-{\left(\frac{B}{L}\right)}^{2}}$$ (23)

**FIGURE 4:**
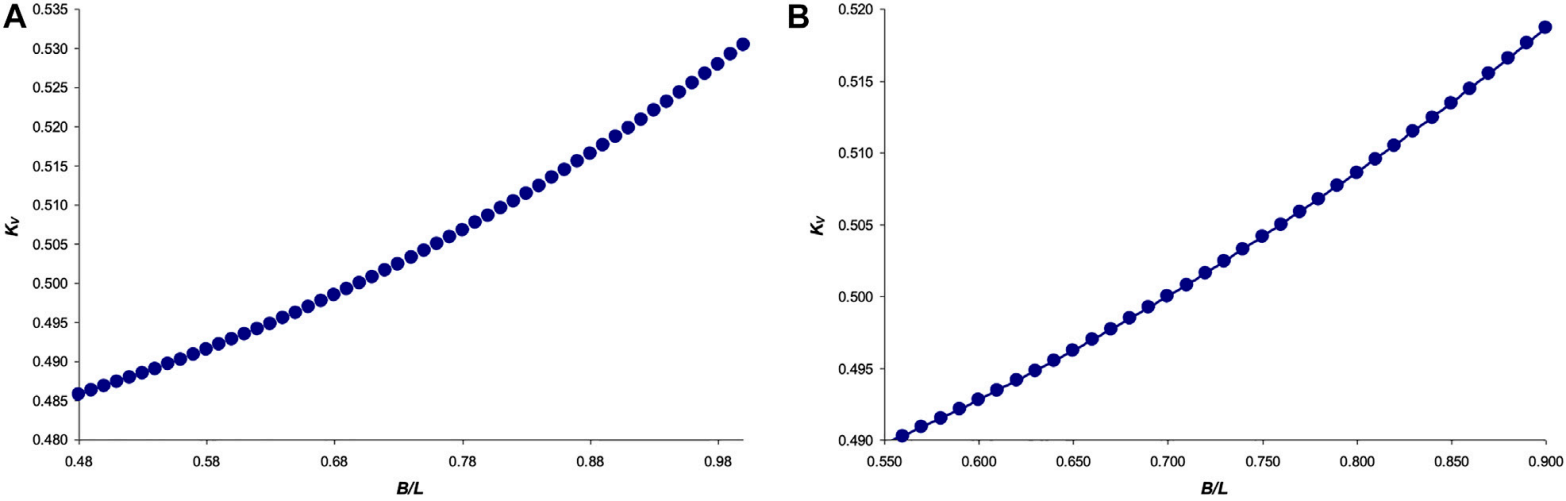
Graphical dependence of the function *K*_*V*_ = *f* (*B*/*L*) for the interval: **(A)** *B*/*L* = [0.48 … 1]; and **(B)** *B*/*L* = [0.55 … 0.90].

Next, we represented Eq. 23 graphically, in the range of values *B*/*L* = [0.48 … 1] (Figure 4A), as well as a slightly narrower interval *B*/*L* = [0.55 … 0.90], more typical for bird eggs (Figure 4B).

The graphical dependence in Figure 4A was fairly accurately approximated by the following equation:

$$K_{V}=0.072{\left(\frac{B}{L}\right)}^{2}-0.021\frac{B}{L}+0.479$$ (24)

*R* ^2^ = 0.9999, and for the dependence in Figure 4B:

$$K_{V}=0.07{\left(\frac{B}{L}\right)}^{2}-0.02\frac{B}{L}+0.48,$$ (25)

*R* ^2^ = 0.99999, which we accepted as the basis of the final formula for calculating *V*.

Therefore, the value of *V* can be calculated by the following final formula:

$$V=\left(0.07{\left(\frac{B}{L}\right)}^{2}-0.02\frac{B}{L}+0.48\right)LB^{2}$$ (26)

Thus, the implemented transformations made it possible to bring Eqs 21 to a different form that is likely to be more suitable for further analysis. We also demonstrated the mathematical nature of *K*_*V*_ variations in the form of a square polynomial.

For simpler transformations, which we would need in the subsequent analysis, we also used a linear approximation. Although the latter could somewhat reduce the accuracy, it was extremely useful in determining the internal parameters of the ovoid shell. In this case,

$$K_{V}=0.083\frac{B}{L}+0.443$$ (27)

*R* ^2^ = 0.994, and, correspondingly,

$$V=\left(0.083B+0.443L\right)B^{2}$$ (28)

To derive *S*, we employed the classical integral geometry formula, resulting in the following final expression:

$$S=\frac{2\pi \left(0.0705n+0.9149\right)}{n+1}L^{2}$$ (29)

Detailed output of Eq. 29 is presented in Supplementary Material S1.

After substituting Eq. 13 into Eq 29, we obtained:

$$S=\frac{0.44\left({\left(\frac{L}{B}\right)}^{2}+8.49\right)}{{\left(\frac{L}{B}\right)}^{2}+0.35}L^{2}$$ (30)

To make Eq. 30 more convenient for subsequent analysis and mathematical transformations, we performed the same operations as we did for the volume calculation formula (Eq. 22). Let us rewrite Eq. 30 as follows:

$$S=K_{S}L^{2}$$ (31)

where the coefficient *K*_*S*_, respectively, is equal to

$$K_{S}=\frac{0.44\left({\left(\frac{L}{B}\right)}^{2}+8.49\right)}{{\left(\frac{L}{B}\right)}^{2}+0.35}$$ (32)

**FIGURE 5:**
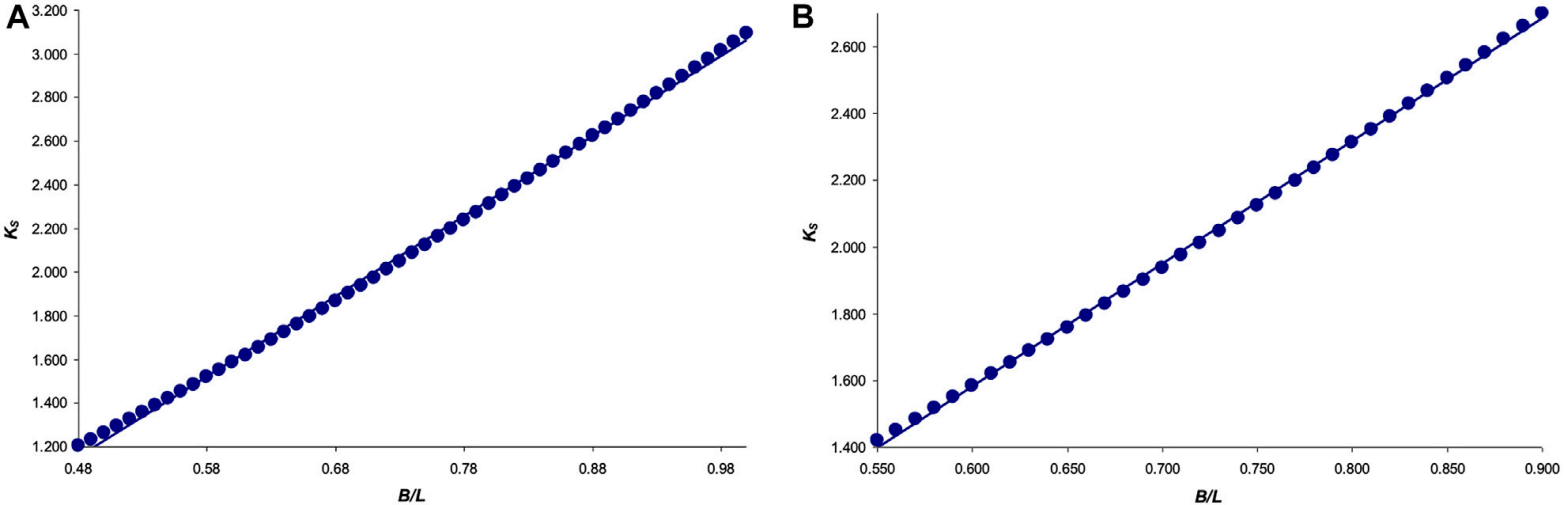
Graphical dependence of the function *K*_*S*_ = *f* (*B*/*L*) for the interval: **(A)** *B*/*L* = [0.48 … 1]; and **(B)** *B*/*L* = [0.55 … 0.90].

To represent it graphically, we used the restricted range of values *B*/*L* = [0.48 … 1] (Figure 5A), as well as a somewhat narrower interval *B*/*L* = [0.55 … 0.90], more typical for the whole variety of bird eggs (Figure 5B).

The graphical dependence in Figure 5A was fairly accurately approximated by the following equation:

$$K_{S}=3.67\frac{B}{L}-0.61$$ (33)

$$R^{2}=0.999\text{,}$$

and that in Figure 5B:

$$K_{S}=3.68\frac{B}{L}-0.62$$ (34)

$$R^{2}=0.999\text{,}$$

which we used further as the basis of the formula for calculating *S*. That is, we finally had the following equation:

S=(3.68B−0.62L)L (35)

### Calculation of the internal parameters of an ovoid described using Narushin’s model

Having the calculation formulae for *V* (Eq. 28) and *S* (Eq. 35), we proceeded to calculate the volumes of the interior (internal contents), *V*_*i*_, and shell, *V*_*s*_, as well as the location of the neutral axis within the shell, if it has the shape following Narushin’s model.

The volume of the contents, *V*_*i*_, in the case of ovoid shell implies the following calculation of this parameter along the blue line in Figure 2, taking into account the fact that the linear dimensions will decrease by twice the shell thickness, *T*:

$$V_{i}=\left(0.083\left(B-2T\right)+0.443\left(L-2T\right)\right){\left(B-2T\right)}^{2}=V-T\left(1.052B^{2}+4\left(B+T\right)\left(0.083B+0.443L-1.052T\right)\right)$$ (36)

Considering Eq. 5, it can be argued that the shell volume, *V*_*s*_, in our case is equal to

$$V_{s}=T\left(1.052B^{2}+4\left(B+T\right)\left(0.083B+0.443L-1.052T\right)\right)$$ (37)

Another approach to calculating *V*_*s*_ is to use Eq. 6. On the one hand, the value of *S*_*n*_ can be computed by dividing Eq 37 by *T*:

$$S_{n}=1.384B^{2}+1.772BL-3.876BT+1.772LT-4.208T^{2}$$ (38)

Also, the formula for calculating *S*_*n*_ (purple line in Figure 2) can be deduced by reducing the geometric dimensions in Eq. 35 by 2*kT*. These changes resulted in the following expression:

$$S_{n}=12.24k^{2}T^{2}-4.88kLT-7.36kBT+3.68BL-0.62L^{2}$$ (39)

Let us equate Eqs 38, 39 and reconsider for *k*. As a result of solving the quadratic equation, we obtained:

$$k=0.2\frac{L}{T}+0.3\frac{B}{L}\cdot \frac{L}{T}-\sqrt{0.091{\left(\frac{L}{T}\right)}^{2}+0.203{\left(\frac{B}{L}\right)}^{2}\cdot {\left(\frac{L}{T}\right)}^{2}-0.036\left(\frac{B}{L}\right){\left(\frac{L}{T}\right)}^{2}-0.317\frac{B}{L}\cdot \frac{L}{T}+0.145\frac{L}{T}-0.344}$$ (40)

### Comparative analysis of Narushin’s and Hügelschäffer’s models

Having the entire suite of calculation formulae for both models available, it was possible to carry out a comparative analysis using the five steps indicated in the Materials and methods section and the appropriate Eqs. 5–9. As an initial dataset, we used the data typical for the standard chicken egg as proposed on the basis of numerous measurements in (Romanoff and Romanoff, 1949), i.e., *L* = 5.7 cm, and *B* = 4.2 cm. The average shell thickness can be accepted equal to 0.034 cm, which, in addition to our previous measurements (Narushin, 2001a), was also confirmed in other studies (Lichovnikova, 2007; Liao et al., 2013; Ketta and Tůmová, 2018). The choice of such standard chicken egg is by no means a recommendation for manufacturing engineering and building structures with a similar ratio of parameters. This was due only to the desire of the authors to choose a model object that is well known to everyone and visually represented. The comparison results are given in Table 1.

**TABLE 1:** Results of comparative analysis of egg parameters using Narushin’s and Hügelschäffer’s models.

Variable	Hügelschäffer’s Model		Narushin’s Model	
	Calculative Formula	Result	Calculative Formula	Result
Length, *L*, cm	**–**	5.7	**–**	5.7
Maximum breadth, *B*, cm	**–**	4.2	**–**	4.2
Shell thickness, *T*, cm	**–**	0.034	**–**	0.034
Vertical axis shift, *w*, cm	Eq 18	0.28	Eq 18	0.28
Volume, *V*, cm^3^	Narushin et al. (2022c)	52.46	Eq 21	50.58
Surface area, *S*, cm^2^	Narushin et al. (2022c)	68.98	Eq 30	67.39
Shell volume, *V*_*s*_, cm^3^	Eq 7	2.30	Eq 37	2.27
Volume of the interior, *V*_*i*_,cm^3^	Eqn 8	50.16	Eq 36	48.31
Position of the neutral axis, *k*	Eq 9	0.713	Eq 40	0.627

### Optimization of *k* values

As follows from Table 1, the neutral axis in Narushin’s model is slightly higher towards the outer surface than in Hügelschäffer’s model. In this regard, if the hypothesis of the strength of thin-walled shells is adequate, an ovoid made in accordance with Eq. 1 has some advantage over a similar ovoid according to Eq. 4, with other geometric and design parameters being equal. Therefore, it would be highly useful to investigate the functional changes in the value of *k* in Eq. 40. Similar studies were carried out in our previous work (Narushin et al., 2022c) for Hügelschäffer’s model, i.e., for Eq. 9, as a result of which the following patterns were revealed: the value of *k* decreases (i) with an increase in the *B*/*L* ratio, i.e., when the ovoid tends to a spherical shape, and also (ii) with a decrease in the ratio *L*/*T*, i.e., when there is an increase in the shell thickness in comparison with the ovoid length. These results obtained were quite convincing and expected. However, in the practical realm, we are limited by the design expediency, and therefore, we cannot allow an increase in thickness, as well as the transformation of an egg shape into a spherical one. We have already accepted a priori that we are interested in the bionic relationship of an actual bird’s egg and its artificial counterpart with the maximum duplication in ratios of geometric dimensions.

**FIGURE 6:**
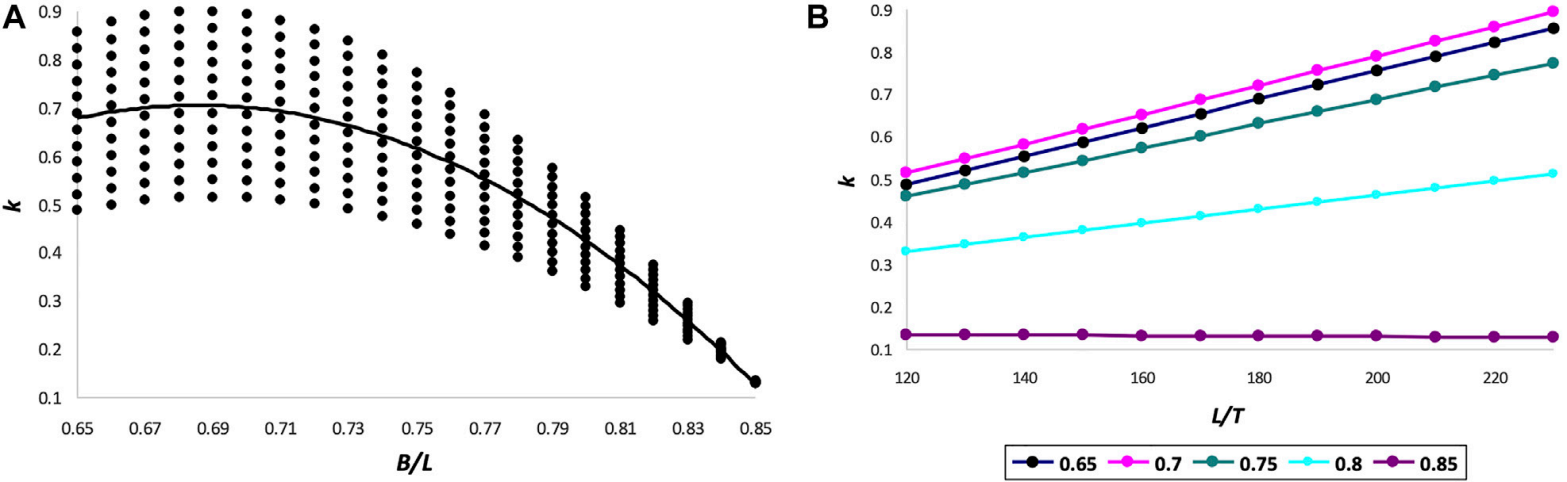
Functional dependences for changes in the value of *k* by *B*/*L* **(A)** and by *L*/*T* **(B)**.

In this regard, when analyzing Eq. 40, we limited ourselves to the ratios of the variables included in this equation, which are characteristic of bird eggs. By analogy with the choice of these ratios and their validation, as described elsewhere (Narushin et al., 2022c), we used the following intervals: *L*/*T* = [120 … 230], and *B*/*L* = [0.65 … 0.85]. Substituting all possible values of *L*/*T* and *B*/*L* from the indicated intervals, we generated their 108 different combinations, which, after substitution into Eq. 40, led to the corresponding values of *k*. Functional dependencies *k* = *f* (*L*/*T*) and *k* = *f* (*B*/*L*) are graphically illustrated in Figure 6.

## Discussion

In practice, the anticipated use of ovoids made in accordance with the mathematical model (Eq. 1), which we named Narushin’s model, necessitated a more thorough overview and analysis of it. It is very likely that this model may be the best choice in terms of borrowing the structures of natural objects for the needs of the engineering and construction industries. While the similar Hügelschäffer’s model has been examined quite comprehensively, Narushin’s model is still understudied in this respect. However, in relation to the fact that only two geometric dimensions, length and maximum breadth, are sufficient for its design, Narushin’s model has certain convenient prerequisites for its preferable use.

After conducting the theoretical research, we were able to establish the main relationships of geometric dimensions for Narushin’s model, clarify the formulae to compute the volume (Eq. 21) and surface area (Eq. 30) for the body of revolution of this figure. Also, the use of Narushin’s model enabled to calculate internal parameters, including the volume of contents (Eq. 36), shell volume (Eq. 37), and, importantly, the location of the neutral axis along the thickness of the shell of an egg-shaped structure (Eq. 40).

Compared to a Hügelschäffer’s model-derived ovoid, Narushin’s model provided a smaller volume of contents, whereas it also showed a correspondingly lower cost for manufacturing the walls of ovoid vessel. If we used the conditional indicator of shell material consumption per unit of internal volume, *V*_*s*_/*V*_*i*_, it turned out, in the framework of our example, to be quite similar to one another, with a slight advantage of Hügelschäffer’s model (0.046) over Narushin’s one (0.047). However, this advantage is easily eliminated by rational selection of geometric parameters.

The examination results of the neutral axis location turned out to be more favorable, because it, according to our hypothesis, provides a greater margin of strength for the thin-walled shell structure. In the Narushin’s model ovoid, the neutral axis is located closer to the surface, which can furnish this design with some advantages in the event of external impact.

A more thorough investigation of the effect of the ovoid’s geometric parameters on the neutral axis location enabled to establish dependencies, the mathematical nature of which differs from those obtained by us for the Hügelschäffer’s model (Narushin et al., 2022c). A particularly interesting observation occurred when the main geometric parameters of Narushin’s ovoid, *B*/*L*, were increased to 0.85 (Figure 4A). In this case, even the minimum shell thickness, *T*, relative to the ovoid length, *L* (Figure 4B), warrants the smallest possible and practically unchanged values of *k*. That is, with this ratio, the neutral axis is located as close as possible to the surface of a thin-walled vessel, thus providing the possibility of its maximum allowable displacement inside the shell structure in the event of external impact.

Generally speaking, it is not always the objective of developing thin-walled structures to secure their strength under external impact. Often, the opposite goals are pursued, i.e., protection from internal pressure. In this case, the displacement of the neutral axis will be directed from the inner to the outer surface. Therefore, for these purposes, one should choose geometric parameters that warrant the maximum value of the *k*-factor. This is achievable with *B*/*L* values around 0.7 (Figure 4A). Herewith, even a decrease in shell thickness leads to an increase in *k* (Figure 4B).

Taking the above into account, we would like to emphasize that the present study is theoretical in nature. The obtained calculation formulae and the analyzed data will undoubtedly facilitate narrowing the range of practical experiments, thereby optimizing the costs of their implementation.

## Conclusion

Narushin’s model may be preferable for using as a mathematical basis for the design of thin-walled shells and a number of building structures. This is due to its relative simplicity, in view of the presence of only two initial parameters in the basic equation, rather optimal geometry in terms of material costs per unit of internal capacity, and effective prerequisites for the strength characteristics of the shell. At the same time, the demonstrated functional changes in the ratios of geometric parameters, *B*/*L* and *L*/*T*, provide an extended toolbox of possible variations when creating engineering and building structures, depending on specific needs, i.e., capacity, strength, load application points, etc.

## Data Availability

The original contributions presented in the study are included in the article/Supplementary Material. Further inquiries can be directed to the corresponding authors.
