# Supplementary material for: Egg-inspired engineering in the design of thin-walled shelled vessels: a theoretical approach for shell strength
Source: Front Bioeng Biotechnol. 2022 Sep 14;10:995817. doi: 10.3389/fbioe.2022.995817 (PMC9516309; doi:10.3389/fbioe.2022.995817)
Supplement: Supplementary file 1 [file DataSheet1.doc]

**Appendix A. Supplementary data**

In accordance with the classical formula of integral geometry, the surface area of a body of revolution in our case is determined by the following formula:

$S=2\pi\int_{0}^{L} y\sqrt{1+\left(\frac{\mathrm{d}y}{\mathrm{d}x}\right)^{2}}\mathrm{d}x$ (S1)

First, let us define the derivative of the Narushin’s model (Eq. (1)) function:

$$\frac{\mathrm{d}y}{\mathrm{d}x}=\frac{1}{2\sqrt{L^{\frac{2}{n+1}}x^{\frac{2n}{n+1}}-x^{2}}}\left(L^{\frac{2}{n+1}}\frac{2n}{n+1}x^{\frac{2n}{n+1}-1}-2x\right)=\frac{L^{\frac{2}{n+1}}\frac{2n}{n+1}x^{\frac{2n-n-1}{n+1}}-2x}{2\sqrt{L^{\frac{2}{n+1}}x^{\frac{2n}{n+1}}-x^{2}}}=$$

$$=\frac{L^{\frac{2}{n+1}}nx^{\frac{n-1}{n+1}}-x(n+1)}{(n+1)\sqrt{L^{\frac{2}{n+1}}x^{\frac{2n}{n+1}}-x^{2}}}=\frac{x\left(L^{\frac{2}{n+1}}nx^{\frac{n-1}{n+1}-1}-n-1\right)}{(n+1)x\sqrt{L^{\frac{2}{n+1}}x^{\frac{2n}{n+1}-2}-1}}=\frac{L^{\frac{2}{n+1}}nx^{\frac{n-1-n-1}{n+1}}-n-1}{(n+1)\sqrt{L^{\frac{2}{n+1}}x^{\frac{2n-2n-2}{n+1}}-1}}=$$

$=\frac{L^{\frac{2}{n+1}}nx^{-\frac{2}{n+1}}-n-1}{(n+1)\sqrt{L^{\frac{2}{n+1}}x^{-\frac{2}{n+1}}-1}}$ (S2)

As a result, the integral (Eq. (S1)) will be rewritten in the following form:

$$=2\pi\int_{0}^{L} x\sqrt{L^{\frac{2}{n+1}}x^{-\frac{2}{n+1}}-1}\frac{\sqrt{(n+1)^{2}\left(L^{\frac{2}{n+1}}x^{-\frac{2}{n+1}}-1\right)+\left(L^{\frac{2}{n+1}}nx^{-\frac{2}{n+1}}-n-1\right)^{2}}}{(n+1)\sqrt{L^{\frac{2}{n+1}}x^{-\frac{2}{n+1}}-1}}\mathrm{d}x=$$

$$=\frac{2\pi}{n+1}\int_{0}^{L} x\sqrt{(n+1)^{2}\left(L^{\frac{2}{n+1}}x^{-\frac{2}{n+1}}-1\right)+\left(L^{\frac{2}{n+1}}nx^{-\frac{2}{n+1}}-n-1\right)^{2}}\mathrm{d}x=$$

$$=\frac{2\pi}{n+1}\int_{0}^{L} x\sqrt{(n+1)^{2}\left(L^{\frac{2}{n+1}}x^{-\frac{2}{n+1}}-1\right)+\left(n\left(L^{\frac{2}{n+1}}x^{-\frac{2}{n+1}}-1\right)-1\right)^{2}}\mathrm{d}x=$$

$$=\frac{2\pi}{n+1}\int_{0}^{L} x\sqrt{(n+1)^{2}\left(L^{\frac{2}{n+1}}x^{-\frac{2}{n+1}}-1\right)+n^{2}\left(L^{\frac{2}{n+1}}x^{-\frac{2}{n+1}}-1\right)^{2}-2n\left(L^{\frac{2}{n+1}}x^{-\frac{2}{n+1}}-1\right)+1}\mathrm{d}x=$$

$$=\frac{2\pi}{n+1}\int_{0}^{L} x\sqrt{n^{2}\left(L^{\frac{2}{n+1}}x^{-\frac{2}{n+1}}-1\right)^{2}+(n^{2}+2n+1-2n)\left(L^{\frac{2}{n+1}}x^{-\frac{2}{n+1}}-1\right)+1}\mathrm{d}x=$$

$$=\frac{2\pi}{n+1}\int_{0}^{L} x\sqrt{n^{2}\left(L^{\frac{2}{n+1}}x^{-\frac{2}{n+1}}-1\right)^{2}+(n^{2}+1)\left(L^{\frac{2}{n+1}}x^{-\frac{2}{n+1}}-1\right)+1}\mathrm{d}x=$$

$$=\frac{2\pi}{n+1}\int_{0}^{L} x\sqrt{n^{2}\left(L^{\frac{2}{n+1}}x^{-\frac{2}{n+1}}-1\right)^{2}+n^{2}\left(L^{\frac{2}{n+1}}x^{-\frac{2}{n+1}}-1\right)+L^{\frac{2}{n+1}}x^{-\frac{2}{n+1}}-1+1}\mathrm{d}x=$$

$$=\frac{2\pi}{n+1}\int_{0}^{L} x\sqrt{n^{2}\left(L^{\frac{2}{n+1}}x^{-\frac{2}{n+1}}-1\right)\left(L^{\frac{2}{n+1}}x^{-\frac{2}{n+1}}-1+1\right)+L^{\frac{2}{n+1}}x^{-\frac{2}{n+1}}}\mathrm{d}x=$$

$$=\frac{2\pi}{n+1}\int_{0}^{L} x\sqrt{n^{2}L^{\frac{2}{n+1}}x^{-\frac{2}{n+1}}\left(L^{\frac{2}{n+1}}x^{-\frac{2}{n+1}}-1\right)+L^{\frac{2}{n+1}}x^{-\frac{2}{n+1}}}\mathrm{d}x=$$

$$=\frac{2\pi}{n+1}\int_{0}^{L} x\sqrt{L^{\frac{2}{n+1}}x^{-\frac{2}{n+1}}\left(n^{2}\left(L^{\frac{2}{n+1}}x^{-\frac{2}{n+1}}-1\right)+1\right)}\mathrm{d}x=$$

$$=\frac{2\pi}{n+1}\int_{0}^{L} xL^{\frac{1}{n+1}}x^{-\frac{1}{n+1}}\sqrt{n^{2}\left(L^{\frac{2}{n+1}}x^{-\frac{2}{n+1}}-1\right)+1}\mathrm{d}x=$$

$$=\frac{2\pi}{n+1}\int_{0}^{L} x\left(\frac{L}{x}\right)^{\frac{1}{n+1}}\sqrt{n^{2}\left(\left(\frac{L}{x}\right)^{\frac{2}{n+1}}-1\right)+1}\mathrm{d}x=$$

$$=\frac{2\pi}{n+1}\int_{0}^{L} x\cdot\frac{L}{L}\cdot\left(\frac{L}{x}\right)^{\frac{1}{n+1}}\sqrt{n^{2}\left(\left(\frac{L}{x}\right)^{\frac{2}{n+1}}-1\right)+1}\mathrm{d}x=$$

$$=\frac{2\pi L}{n+1}\int_{0}^{L} \frac{x}{L}\cdot\left(\frac{x}{L}\right)^{-\frac{1}{n+1}}\sqrt{n^{2}\left(\left(\frac{x}{L}\right)^{-\frac{2}{n+1}}-1\right)+1}\mathrm{d}x=$$

$$=\frac{2\pi L}{n+1}\int_{0}^{L} \left(\frac{x}{L}\right)^{1-\frac{1}{n+1}}\sqrt{n^{2}\left(\left(\frac{x}{L}\right)^{-\frac{2}{n+1}}-1\right)+1}\mathrm{d}x=\frac{2\pi L}{n+1}\int_{0}^{L} \left(\frac{x}{L}\right)^{\frac{n+1-1}{n+1}}\sqrt{n^{2}\left(\left(\frac{x}{L}\right)^{-\frac{2}{n+1}}-1\right)+1}\mathrm{d}x=$$

$=\frac{2\pi L}{n+1}\int_{0}^{L} \left(\frac{x}{L}\right)^{\frac{n}{n+1}}\sqrt{n^{2}\left(\left(\frac{x}{L}\right)^{-\frac{2}{n+1}}-1\right)+1}\mathrm{d}x$ (S3)

Let us make a change of variable in the integral of Eq. (S3) and assume

$t=\frac{x}{L}$ (S4)

Then,

$\text{d}x=L\text{d}t$ (S5)

Let us set new limits of integration:

$$x_{\min}=0$$

Hence,

$$t_{\min}=\frac{0}{L}=0$$

$$x_{\max}=L$$

Then,

$$t_{\max}=\frac{L}{L}=1$$

Based on the above, Eq. (S3) will receive the form:

$S=\frac{2\pi L}{n+1}\int_{0}^{1} t^{\frac{n}{n+1}}\sqrt{n^{2}\left(t^{-\frac{2}{n+1}}-1\right)+1}\cdot L\mathrm{d}t=\frac{2\pi L^{2}}{n+1}\int_{0}^{1} t^{\frac{n}{n+1}}\sqrt{n^{2}\left(t^{-\frac{2}{n+1}}-1\right)+1}\mathrm{d}t$ (S6)

In the resulting Eq. (S6), we will separately consider the integral directly, which we denote as *I*:

$I=\int_{0}^{1} t^{\frac{n}{n+1}}\sqrt{n^{2}\left(t^{-\frac{2}{n+1}}-1\right)+1}\mathrm{d}t$ (S7)

the integral (Eq. (S7)) was determined by numerical methods in MS Excel (e.g., Piessens et al., 1983) at different values of *n* = [1…6], which corresponds to *B*/*L* = [0.48...1], i.e., any possible natural variations of bird eggs. the obtained data (Supplementary Figure 1) was approximated by the corresponding equation:

**Supplementary Figure 1.** Graphic dependence of the function *I* = *f*(*n*) for the interval *n* = [1…6].

$I=0.0705n+0.9149$ (S8)

As a result, the final equation for calculating *S* will be written as:

$S=\frac{2\pi(0.0705n+0.9149)}{n+1}L^{2}$ (S9)

Let us verify whether the calculation formula for *S* (Eq. (S9)) will change if the *B*/*L* values differ from the interval characteristic of bird eggs.

To do this, let us recalculate the value of *I* (Eq. (28)) in a wider range, say, *n* = [1…100], which corresponds to *B*/*L* = [0.12...1]. The obtained data is shown graphically in Supplementary Figure 2.

**Supplementary Figure 2.** Graphic dependence of the function *I* = *f*(*n*) for the interval *n* = [1…100].

As the value of *n* increases, i.e., with corresponding decrease in the *B*/*L* ratio, the integral dependence (Eq. (S7)) acquires a curvilinear character, which can be approximated by the parabolic equation:

$I=-0.00022n^{2}+0.05577n+1.00793$ (S10)

For such a broader case, the value of *S* is defined in the following form:

$S=\frac{2\pi L^{2}}{n+1}(-0.00022n^{2}+0.05577n+1.00793)=\frac{0.014(4581.5+253.5n-n^{2})}{n+1}L^{2}$ (S11)

However, within the framework of the present investigation, we were more interested in the intervals corresponding specifically to bird eggs, and therefore, Eq. (S9) was chosen as the basis for further mathematical transformations.

**Reference**

Piessens, R., de Doncker-Kapenga, E., Überhuber, C.W., and Kahaner, D.K. (1983). QUADPACK: A Subroutine Package for Automatic Integration. Berlin–Heidelberg–New York: Springer Verlag. https://excel-works.com/manual/quadf [Accessed July 16, 2022].
